# Performance Evaluation of Bundle Adjustment with Population Based Optimization Algorithms Applied to Panoramic Image Stitching

**DOI:** 10.3390/s21155054

**Published:** 2021-07-26

**Authors:** Maria Júlia R. Aguiar, Tiago da Rocha Alves, Leonardo M. Honório, Ivo C. S. Junior, Vinícius F. Vidal

**Affiliations:** Department of Electrical Engineering, Federal University of Juiz de Fora, Juiz de Fora 36036-900, Brazil; maria.aguiar@engenharia.ufjf.br (M.J.R.A.); tiago.alves@engenharia.ufjf.br (T.d.R.A.); ivo.junior@ufjf.edu.br (I.C.S.J.); vinicius.vidal@engenharia.ufjf.br (V.F.V.)

**Keywords:** metaheuristics, bundle adjustment, panorama image, bat algorithm, grey wolf optimizer, arithmetic optimization algorithm, Salp Swarm Algorithm, particle swarm optimization

## Abstract

The image stitching process is based on the alignment and composition of multiple images that represent parts of a 3D scene. The automatic construction of panoramas from multiple digital images is a technique of great importance, finding applications in different areas such as remote sensing and inspection and maintenance in many work environments. In traditional automatic image stitching, image alignment is generally performed by the Levenberg–Marquardt numerical-based method. Although these traditional approaches only present minor flaws in the final reconstruction, the final result is not appropriate for industrial grade applications. To improve the final stitching quality, this work uses a RGBD robot capable of precise image positing. To optimize the final adjustment, this paper proposes the use of bio-inspired algorithms such as Bat Algorithm, Grey Wolf Optimizer, Arithmetic Optimization Algorithm, Salp Swarm Algorithm and Particle Swarm Optimization in order verify the efficiency and competitiveness of metaheuristics against the classical Levenberg–Marquardt method. The obtained results showed that metaheuristcs have found better solutions than the traditional approach.

## 1. Introduction

Over the years, several computer vision simulation techniques and methods have been developed to extract information in order to understand the environment through the use of sensors and cameras. As computers and cameras have become more affordable, the use of digital images has grown, and thus generated an increasing interest in high-resolution images. The automatic panoramic image stitching, as an example, is an important field of study for the research community due to its use in several applications in computer vision [1], photogrammetry [2,3,4] and remote sensing [5,6,7,8].

Panoramic images are obtained to produce images with wide field of view (FOV) and depict large objects that cannot be captured in a single image. Therefore, multiple images of the same environment are combined to obtain the whole scene. Image stitching is the process of overlapping a set of images obtained from different points of view, time, visual angles and sensors to generate a high resolution image with a wider view [9,10].

Although seamless panoramic images the desired result for applications, and there are advances in this area of study in recent years, image stitching remains a challenge due to factors such as registration and blending [11]. Some questions remain unresolved and from a computational geometry perspective the result usually contains cut objects or blur [12]. The image stitching method is the joining of images based on their overlapping areas and can be defined in two main steps: alignment and blending [13].

In image registration process, several errors can arise when images are grouped. Geometric and photometric misalignment often result in undesirable discontinuities of objects and visibility in the overlap area between images [14]. However, perfect alignment is rarely achieved. In that regard, most efforts in this research field are focused on designing better alignment or compositing techniques to reduce or hide misalignment. Some algorithms are used in order to minimize the coloration discontinuities in the appearance of the final generated image, being some techniques of pixel matching and image blending [15,16,17,18]. The research on images alignment for stitching culminated, to some extent, in the use of bundle adjustment [19] to simultaneously optimize the relative positions of the images. Several types and classes of optimization-based algorithms [20,21,22,23,24,25,26] can be used to align all images to a common frame of reference typically, the classic Levenberg–Marquardt method is used to optimize the intrinsic and extrinsic parameters of the camera [27,28].

This paper proposes the comparison of the Levenberg-Maquardt algorithm, which is commonly used in the literature, against some meta heuristics that are well proven to be a good solution to multimodal optimization problems, that tend to be affected by local minima [29]. Thus, it aims to experiment a modified optimization approach for the Bundle Adjustment of a custom 360-degree image capture device. Therefore, this research’s main contribution is the comparison of the Levenberg–Marquardt method against bio-inspired algorithms, such as Bat Algorithm, Grey Wolf Optimizer, Salp Swarm Algorithm, Arithmetic Optimization Algorithm and Particle Swarm Optimization in Bundle adjustment optimization of the intrinsic and extrinsic parameters of cameras, focusing on improving the system’s image alignment stage. The remainder of this work is organized as follows: Section 2 details the proposed methodology and its mathematical and construction foundations. Section 3 presents the results and discussions about what was proposed and improved when using bio-inspired meta heuristics. The final concluding remarks and ideas for future works are presented in Section 4.

## 2. Proposed Study Methodology

This paper proposes a comparison of optimization methods for image alignment using bundle adjustment to generate spherical panoramic images. This section presents the overview of the proposed pipeline, followed by the main components of the methodology.

### 2.1. Framework Overview

**Figure 1:**
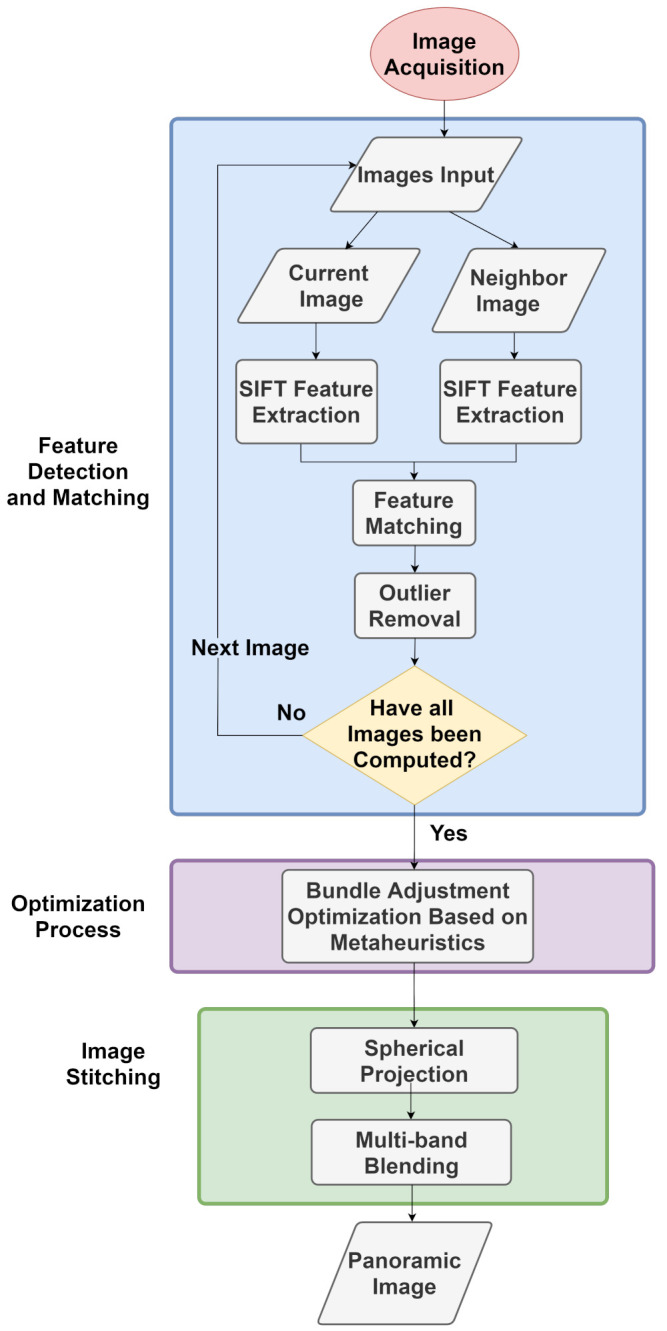
Flowchart of the Proposed Method.

Figure 1 shows the general architecture of the proposed method. The panoramic image generation pipeline is composed of several steps including image acquisition, feature extraction and matching, bundle adjustment, spherical projection, image stitching and blending. The first step is to capture images using a proprietary robotic system consisting of a camera and laser on a mechanical structure capable of rotation in pan and tilt directions under controlled conditions, which are used as input data for the system. Feature points are extracted from the images and combined with each other, resulting in a set of matched pairs. In the next step, the pairs are used to obtain the intrinsic and extrinsic parameters of the images by bundle adjustment, in which the re-projection error of the correspondences is minimized using optimization. Finally, a spherical projection is performed, as each image is projected onto a spherical surface and all image are stitched and blended together to obtain the final panorama. The components of the methodology are described in separated sections with detailed explanation.

### 2.2. Data Acquisition

**Figure 2:**
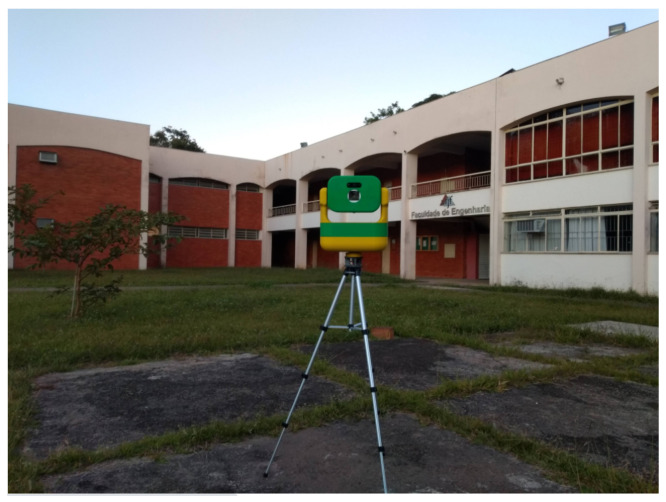
Data Acquisition System.

Image acquisition is performed using a robotic system illustrated in Figure 2. The robot is a scanning system capable of obtaining the reconstruction of an entire 3D environment or object that can be used in augmented and virtual reality applications. This concept inserts a camera and a laser into a mechanical structure that is capable of rotating under controlled conditions. It is composed of a Livox MID-40 LIDAR laser scanner, a Logitech C925e USB camera, two Dynamixel AX-18A servo motors and an InvenSense Intertial Measurement Unit (IMU) MPU-6050. The control strategy is a fault tolerant nonlinear feedback linearization [30,31,32]. This helps with image reconstruction, as the acquisition poses are previously known using the initial orientations from the IMU and servo angles as references in the Bundle Adjustment process. The angle positioning and intrinsic parameter information of each camera are included in the respective datasets. The acquired images are available in [33].

This module was used to acquire several high-resolution images for assembling 360° panoramas, using distance and positioning data from IMU and servo motors angles. This would be essential to ensure that the scene of interest is effectively recorded.

The servo motors are responsible for pan and tilt movements over a range of 360 and 120 degrees, respectively. The images were taken using a 50° pan and 15° tilt step to move the servomotor, covering the 360° region. In this way, sets of 56 images from the Arts and Design School at Federal University of Juiz de Fora were acquired. For this work, 1280 × 720 pixels HD image sets were used.

### 2.3. Feature Detection and Matching

Image alignment consists of overlapping two or more images of the same scene taken at different times, establishing geometric correspondences between pairs of images that represent the same environment [34]. In order, to register multiple images, it is necessary to estimate the transformations that align them according to an image reference within the data set. Therefore, the images are aligned to a common coordinate using the computed geometric transformation. The aligned images are overlaid on a larger image by merging the pixel values of the overlap area so that the border is continuous with a smooth transition between images.

The first step in the alignment process is to extract and find correspondences between images. The Scale Invariant Feature Transform (SIFT) algorithm developed by Lowe [35] was used for this method and is widely used in image stitching [17,36,37,38] . After calculating the descriptors for all images, points are found in the images that have similar descriptors. For each feature found, the two best candidate matches (nearest matches) were maintained. To improve the match set, the ratio test is first applied according to (1) in which $d_{1}$ is the nearest neighbor distance, $d_{2}$ is the second nearest neighbor distance and the ratio value less than a threshold (θ) set to 0.8 is considered a good match. After this condition, unwanted matches are discarded. Even so, in the resulting correspondences there can still be found outliers. Therefore, the good matches are filtered by calculating the fundamental matrix, capable of mapping points from one image to another with the Random Sample Consensus Algorithm (RANSAC) [39].

(1) $$\frac{d_{1}}{d_{2}}\leq \theta$$

### 2.4. Optimization Process

After obtaining the set of geometrically consistent matches between images, bundle adjustment is used to solve all camera parameters together. The initial intrinsic and extrinsic parameters for each camera are known. The extrinsic parameters are derived from servo motors positions and the IMU during image acquisition obtaining rotation angles of each image. The focal length and optical centers were previously found by camera calibration. From this structured acquisition information it was possible to find the best neighbors of each image and perform image-matching with the best neighbors. In this step, the optimization process begins. The images are added with their correspondent neighborhood, and the bundle adjustment process is initialized with the values of neighbor’s matches, focal length, optical center and pan and tilt angles for each camera. Then the parameters are update using Bio-inspired methods.

Bio-inspired techniques were utilized in order to verify which method finds the best result for the Bundle Adjustment problem, and compared with the classic Levenberg-Maquardt. Population based metaheuristics were used and some solutions are modified at each iteration, while others reach the next iteration. The modifications of the solution, generally, are made by specific properties of each algorithm and its populations [40]. For that, optimization algorithms as Bat Algorithm, Grey Wolf Optimizer, Salp Swarm Algorithm, Arithmetic Optimization Algorithm and Particle Swarm Optimization were used.

#### 2.4.1. Mathematical Formulation

To solve the camera positioning problem, the approach of relating images according to the homography matrix between images pairs to project them onto a reference plane was used [17]. The homography matrix that represents the pairs between the *i*-th and *j*-th images is given by Equation (2).

(2) $$H_{ij}=K_{i}R_{i}R_{j}^{T}K_{j}^{-1}$$

where

(3) $$K_{i}=\left[\begin{array}{ccc} f_{x} & 0 & c_{x}\\ 0 & f_{y} & c_{y}\\ 0 & 0 & 1 \end{array}\right]$$

represents the intrinsic matrix, $f_{x}$, $f_{y}$ are the focal length and $c_{x}$, $c_{y}$ the optical centers of the camera. The rotation matrix is given by: $R_{i}=R_{\theta i}R_{\phi i}$ where θ and ϕ are the pan and tilt angles, respectively.

Each feature is projected onto all the images it corresponds to, and the sum of the squared distances of images is minimized in relation to the camera parameters. For each image, the parameters representing pan and tilt can be optimized [41], as well as the values of camera’s focal length and optical center. Equation (4) represents the projection residue of *k*-th feature in one image corresponding to the *m*-th feature in another image.

(4) $$r_{ij}^{k}=u_{i}^{k}-p_{ij}^{k}$$

where $u_{i}^{k}$ represents the *k*-th feature in the *i*-th image, $r_{ij}^{k}$ is the residue of the projection of the *k*-th feature of the *j*-th image in *i*-th image, and $p_{ij}^{k}$ is the projection of the image *j* to image *i* of the corresponding point $u_{j}^{m}$, represented by Equation (5b).

(5a) $$p_{ij}^{k}=K_{i}R_{i}R_{j}^{T}K_{i}^{-1}u_{j}^{m}$$

(5b) $$p_{ij}^{k}=H_{ij}u_{j}^{m}$$

Therefore, each camera is defined by rotation ($\phi_{i},\theta_{i}$), focal length ($f_{i}$) and optical centers ($c_{i}$). Thus, the cost function is given by the error represented by the squared sum of the residual errors of all images, presented by Equation (6).

(6) $$C\left({\left\{(\phi_{i},\theta_{i},f_{i},c_{i})\right\}}_{i=1}^{N}\right)=\sum_{i=1}^{N}\sum_{j\in I(i)}\sum_{k\in F(i,j)}{\left(r_{ij}^{k}\right)}^{2}$$

*N* is the number of images, I(i) is the set of images corresponding to the image *i* and F(i,j) is the set of features between images *i* and *j*. The problem consists in minimizing the Equation (6). The parameters values will be found using optimization methods based on bio-inspired algorithm composed of a set of constraints related to the minimum and maximum boundary values of the parameters. Thus, the optimization problem mathematical model can be described according to Equations (7) and (8).

(7) $$Minimize\;fitness=C({\left\{(\phi_{i},\theta_{i},f_{i},c_{i})\right\}}_{i=1}^{N})$$

Subject to:

(8) $$\begin{array}{ccc} \phi_{i}^{min}\leq & \phi_{i}\leq & \phi_{i}^{max}\\ \theta_{i}^{min}\leq & \theta_{i}\leq & \theta_{i}^{max}\\ f_{i}^{min}\leq & f_{i}\leq & f_{i}^{max}\\ c_{i}^{min}\leq & c_{i}\leq & c_{i}^{max} \end{array}$$

As originally conceived, this problem is unrestricted. This represent a problem to population based algorithms as the search space is too wide. In the optimization process, the values obtained during image capture by the robotic system were taken as references for the parameters to be adjusted. In order to address the fact that data provided by the acquisition device might have errors that can cause misalignment, the bounds described in Equation (8) is used to accommodate the sensors’ uncertainty and errors. Thus, these bounds that constrain the parameters between a minimum and maximum value, for this solution, were established empirically. Through tests, the limits for the the rotation angles were set in a range of +5 and −5 degrees of the original acquisition values and focal length and optical centers limited by +5 and −5 pixels from calibration values.

The feasibility of the solutions in metaheuristic methods can be assured in one of two ways: objective function cost penalization for unfeasible generated solutions or, restricting unfeasible generated solutions that surpasses a bound to its value. In this particular study, the latter form is used in order to maintain the population of solutions in a limited search space thus, ensuring the feasibility of the solutions.

#### 2.4.2. Bat Algorithm

As proposed by Yang [42], Bat Algorithm (BA) is a metaheuristic optimization algorithm based on the echolocation behavior of bats, in which bats emits loud pulses in varying frequencies to detect and seek preys or to avoid hitting objects. In that matter, a mathematical analogy was developed to implement it as an optimization problem solving algorithm. To control exploration and exploitation process, the frequency (*f*) and loudness amplitude (*A*) parameters are modified during the iterative process according to (9) and (10). The α parameter represents the decrease rate of amplitude and β is a random vector in the interval [0, 1].

(9) $${fr}_{i}={fr}_{min}+\left({fr}_{max}-{fr}_{min}\right)\beta$$

(10) $$A_{i}^{t+1}=\alpha A_{i}^{t}$$

The positions ($X_{i}^{t}$) and velocities ($V_{i}^{t}$) of the bats are described from Equations (11)–(13).

(11) $$V_{i}^{t+1}=V_{i}^{t}+\left(X_{i}^{t}-X_{*}^{t}\right){fr}_{i}$$

(12) $$X_{i}^{t+1}=X_{i}^{t}+V_{i}^{t+1}$$

For the local exploitation stage, a new bat is generated according to a solution selected among the current best solutions, using a random walk as described in Equation (13).

(13) $$X_{i}^{t+1}=X_{*}^{t}+\epsilon \cdot mean\left(A_{i}^{t}\right)$$

Also, the pulse emission rate increases once a bat is getting closer to a prey according to (14) in which the λ represents the increase rate.

(14) $$r_{i}^{t+1}=1-e^{\lambda t}$$

The Bat Algorithm pseudocode is given in Algorithm 1.
**Algorithm 1** Bat Algorithm (BA) Pseudocode. 
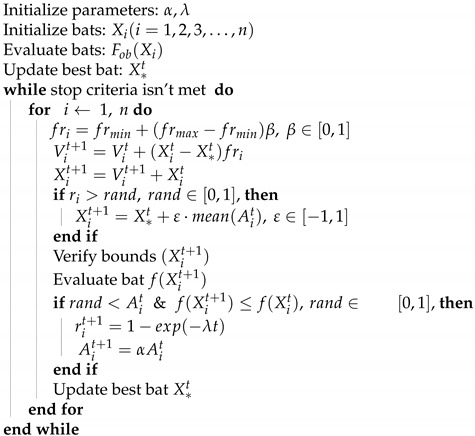


#### 2.4.3. Grey Wolf Optimizer

First published in 2014 [43], the Grey Wolf Optimizer (GWO), is a bio-inspired optimization metaheuristic, based on the hunting behavior of grey wolfs. The algorithm takes into account their social interaction and hierarchy, as well as their approaching pattern when hunting. The wolfs represent possible solutions for the problem and are divided as follows: α the best search agent, β the second best agent, δ the third best agent and ω the rest of the population. The searching process described by a movement of the wolfs according to the α, β and δ positions as described by Equations (15)–(17).

(15) $$\overset{\to }{D_{\alpha }}=\left|\overset{\to }{C_{1}}\cdot \overset{\to }{X_{\alpha }^{t}}-\overset{\to }{X_{i}^{t}}\right|\;\overset{\to }{D_{\beta }}=\left|\overset{\to }{C_{2}}\cdot \overset{\to }{X_{\beta }^{t}}-\overset{\to }{X_{i}^{t}}\right|\;\overset{\to }{D_{\delta }}=\left|\overset{\to }{C_{3}}\cdot \overset{\to }{X_{\delta }^{t}}-\overset{\to }{X_{i}^{t}}\right|$$

(16) $$\overset{\to }{X_{1}}=\overset{\to }{X_{\alpha }^{t}}-\overset{\to }{A_{1}}\cdot \overset{\to }{D_{\alpha }}\;\overset{\to }{X_{2}}=\overset{\to }{X_{\beta }^{t}}-\overset{\to }{A_{2}}\cdot \overset{\to }{D_{\beta }}\;\overset{\to }{X_{3}}=\overset{\to }{X_{\delta }^{t}}-\overset{\to }{A_{3}}\cdot \overset{\to }{D_{\delta }}$$

(17) $${\overset{\to }{X_{i}}}^{t+1}=\frac{\overset{\to }{X_{1}}+\overset{\to }{X_{2}}+\overset{\to }{X_{3}}}{3}$$

The GWO uses a searching parameter *a* to control the exploitation and exploration process, which is decreased linearly from 2 to 0 over the course of the iterations, and is used to limit the fluctuation range of the parameter $\vec{A}$. Note that when the process reaches half of the max iteration value, the wolfs can only move between their position and the position of the prey as $\vec{A}$ is limited by the interval [−1, 1] and the exploitation stage of the algorithm starts.

The Grey Wolf Optimizer pseudocode is given in Algorithm 2.
**Algorithm 2** Grey Wolf Optimizer (GWO) Pseudocode. 
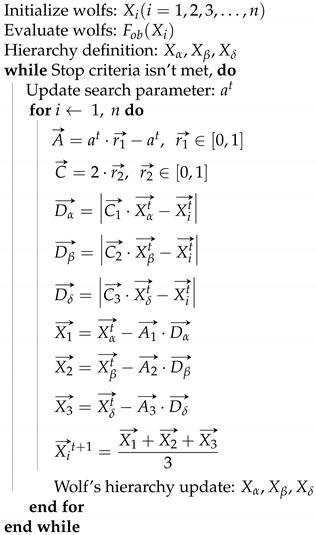


#### 2.4.4. Salp Swarm Algorithm

The Salp Swarm algorithm is a bio-inspired optimization metaheuristic. Firstly published in 2017 [44] and further reviewed in [45], it was inspired by the behavior of salps when navigating and foraging in oceans. As a way to mathematically model the movement of the salp chain, the population is divided into two types: the leader ($X_{i=1}$) and the followers ($X_{i>1}$). The leader is the salp in front of the chain and every other salp is considered as a follower. The best solution obtained so far is modeled as a food source ($X_{*}$) and the position update of the leader is done according to it`s current position as described in Equation (18).

(18) $$\left\{ \begin{array}{c} X_{1}^{t+1}=X_{*}-C_{1}\left[\left(b_{up}-b_{lo}\right).C_{2}+b_{lo}\right]\;C_{3}\leq 0.5\\ X_{1}^{t+1}=X_{*}+C_{1}\left[\left(b_{up}-b_{lo}\right).C_{2}+b_{lo}\right]\;C_{3}>0.5 \end{array}\right.$$

The position update of the follower salps is given by Equation (19).

(19) $$X_{i}^{t+1}=\frac{X_{i}^{t}+X_{i-1}^{t}}{2},\;i\geq 2$$

To control the exploration and exploitation in this method a search parameter $C_{1}$ is proposed. This parameter is updated in each iteration as given by Equation (20).

(20) $$C_{1}=2\cdot exp\left(-{\left(\frac{4t}{t_{max}}\right)}^{2}\right)$$

The Salp Swarm Algorithm pseudocode is given in Algorithm 3.
**Algorithm 3** Salp Swarm Algorithm (SSA) Pseudocode. 
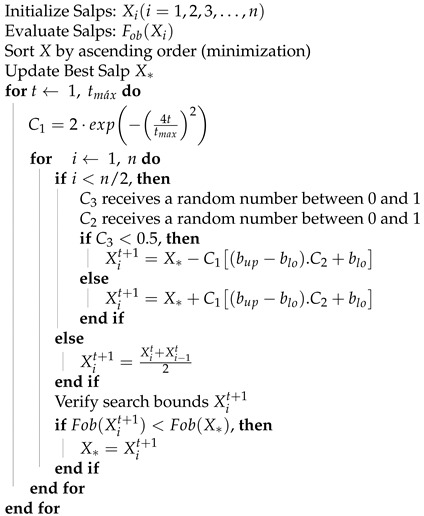


#### 2.4.5. Arithmetic Optimization Algorithm

The Arithmetic optimization algorithm is a novel metaheuristic method that was proposed in 2021 [46]. It is a population based technique and, as the name implies, its based on using Arithmetic operators. The algorithm uses a parameter called Math Optimizer Accelerated (MOA) to control the exploration and exploitation phases as described in (21). Min and Max denotes the maximum and minimum values and are user specified.

(21) $$MOA\left(t\right)=Min\times t\left(\frac{Max-Min}{t_{max}}\right)$$

Another coefficient used in this algorithm is the Math Optimizer probability (MOP) as shown by Equation (22) in which the α denotes a exploitation sensitivity parameter.

(22) $$MOP\left(t\right)=1-\frac{t^{1/\alpha }}{t_{max}^{1/\alpha }}$$

While in the exploration phase, the algorithm uses the Division and Multiplication operators according to Equation (23).

(23) $$x_{i,d}^{t+1}=\{\begin{array}{c} x_{d}^{*}÷\left(MOP+\epsilon \right)\times (\left(X_{*}-C_{1}[\left(b_{d_{up}}-b_{d_{lo}}\right)\times \mu +b_{d_{lo}}\right)\;r_{2}<0.5\\ x_{d}^{*}\times MOP\times (\left(X_{*}-C_{1}[\left(b_{d_{up}}-b_{d_{lo}}\right)\times \mu +b_{d_{lo}}\right)\;\;\;\;r_{2}\;\geq \;0.5 \end{array}$$

The exploitation phase of the algorithm uses the Addition and Subtraction operators and is described in Equation (24).

(24) $$x_{i,d}^{t+1}=\{\begin{array}{c} x_{d}^{*}-MOP\times (\left(X_{*}-C_{1}[\left(b_{d_{up}}-b_{d_{lo}}\right)\times \mu +b_{d_{lo}}\right)\;\;r_{3}<0.5\\ x_{d}^{*}+MOP\times (\left(X_{*}-C_{1}[\left(b_{d_{up}}-b_{d_{lo}}\right)\times \mu +b_{d_{lo}}\right)\;\;r_{3}\geq 0.5 \end{array}$$

The Arithmetic Optimization Algorithm pseudocode is given in Algorithm 4.
**Algorithm 4** Arithmetic Optimization Algorithm (AOA) Pseudocode. 
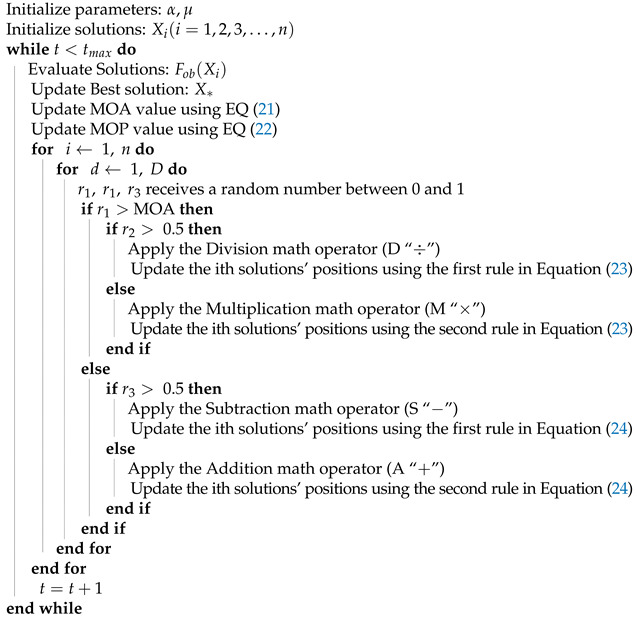


#### 2.4.6. Particle Swarm Optimization

Particle Swarm Optimization (PSO) is a stochastic optimization method based on population developed in 1995 by James Kennedy and Russell Eberhart [47]. The algorithm is based on the collective behavior of flocks of birds and schools of fish by observing the activities of these individuals in dodging predators and searching for food. The initialization of the PSO algorithm occurs with the random creation of the particles that constitute the population (swarm). Particle determination consists of the description of its velocity $\left(V_{\left(i,d\right)}\right)$ and position $\left(X_{i}\right)$ which are expressed through Equations (25) and (26), respectively.

(25) $$V_{\left(i,d\right)}=\omega V_{\left(i,d\right)}+c1\cdot r_{p}\left(X_{\left(i,d\right)}^{p*}-X_{\left(i,d\right)}\right)+c2\cdot r_{g}\left(X_{\left(d\right)}^{g*}-X_{\left(i,d\right)}\right)$$

(26) $$X_{i}=X_{i}+V_{i}$$

As described by Equation (25), the velocity of each particle is updated according to the direction of the individual’s best found position $\left(X_{\left(i,d\right)}^{p*}\right)$ and in the best individual’s found position $\left(X_{\left(d\right)}^{g*}\right)$. ω is a weight parameter that controls the impact of previous particle’s velocity on its current one; $r_{1}$, $r_{2}$ are random variables uniformly distributed between [0, 1] and $c_{1}$, $c_{2}$ are positive constants that control the maximum step size.

The Particle Swarm Optimization pseudocode is given in Algorithm 5.
**Algorithm 5** Particle Swarm Optimization (PSO) Pseudocode. 
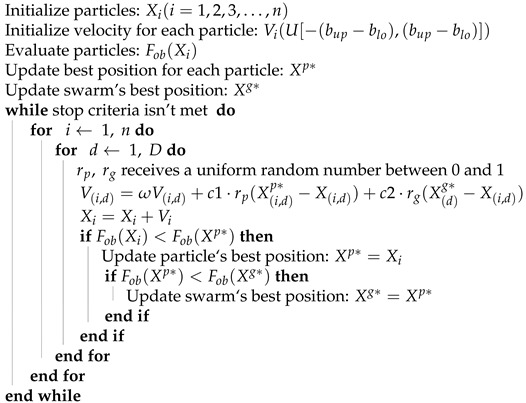


#### 2.4.7. Time Complexity Analysis for the Metaheuristics Algorithms

For the five metaheuristic algorithms focused in this article, the time complexity is dependant on the number of individuals/agents, dimension of the problem and maximum number of iterations. Overall, by analyzing the steps of each algorithm, the computational complexity *t* is stated in Equation (27).

(27) O(t(d·n+C·n))

where *t* is the number of iterations, *d* shows the number of dimensions, *n* indicates the number of search agents and *C* is the cost of objective function.

#### 2.4.8. Levenberg-Maquardt

The Levenberg-Maquardt (LM) Algorithm, first proposed by Kenneth Levenberg [48] and later by Donald Marquardt [49], is a technique that numerically solves nonlinear function minimization problems iteratively by finding local minima of multivariable functions expressed by the sum of squares. This technique is a combination of the gradient descent and Gauss–Newton methods [50] which is based on a local linearization of the residuals according to (28). A vector function *f* maps $p\in R^{m}$ to estimate a measured value $\hat{x}=f\left(p\right)$, $\hat{x}\in R^{n}$. It is desired to minimize the squared distance $\epsilon^{T}\epsilon$ with $\epsilon =x-\hat{x}$ for all **p**, with the initial parameter $p_{0}$ and a measured value x with the intention of at the end finding $p^{+}$ that satisfies the relation *f* locally.

(28) $$f\left(p+\delta_{p}\right)\approx f\left(p\right)+J\delta_{p}$$

(29) $$J=\frac{\partial f(p)}{\partial p}$$

Equation (29) defines the Jacobian matrix of *f* with respect to p. At each iteration a value for $\delta_{p}$ ir estimated in order to minimize Equation (30).

(30) $$||x-f\left(p+\delta_{p}\right)\left|\right|\approx ||x-f\left(p\right)-J\delta_{p}\left|\right|=||\epsilon -J\delta_{p}\left|\right|$$

The $\delta_{p}$ value is the solution of the linear least-squares problem where minimum is found when $J\delta_{p}-\epsilon$ is orthogonal to the column space of J [51]. The LM Algorithm actually solves a equation called as augmented normal equations presented in Equation (31).

(31) $$\left(J^{T}J+\mu I\right)\delta_{p}=J^{T}\epsilon \;\;\mu >0$$

where *I* is the identity matrix, and μ is known as the damping term, which is adjusted at each iteration to ensure error reduction.

The Levenberg-Maquardt pseudocode is given in Algorithm 6.
**Algorithm 6** Levenberg-Maquardt Pseudocode. 
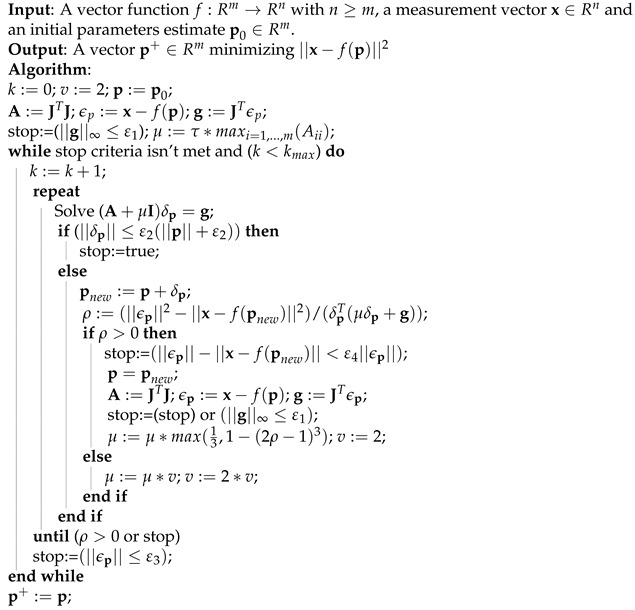


### 2.5. Image Stitching Process

The image stitching process is initialized after adjusting the positioning of the images. First, the spherical projection of the images is performed with the desired final resolution. In this process, the images are mapped on a cartographic plane with a projection formulation that relates pixel and geographic coordinates [52]. The images are projected using the relations ${\left[u,v\right]}^{T}$ = $R{\left[\theta ,\phi \right]}^{T}$, where (θ, ϕ) are the horizontal and vertical angular directions (longitude and latitude, respectively), and (u,v) are pixel coordinates [53]. The projection provides a value (u,v) directly from a value (x,y,z). A point P=(x,y,z) is the 3D projection of a point (x,y) from the original image. Thus, the spherical coordinates can be obtained from the cartesian coordinates given by P, according to the relations shown in (32) and (33):

(32) $$\theta =\arctan\frac{y}{z}$$

(33) $$\phi =\arccos\frac{z}{\sqrt{x^{2}+y^{2}+z^{2}}}$$

In spherical projection, a point can be mapped to a 2D plane simply by setting longitude as horizontal coordinate value and latitude as vertical [54]. After projecting the images there may be lines on the boundaries of the overlapping areas, for example due to the intensity of adjacent pixels differing. Therefore, a correction algorithm is applied to obtain a uniform panorama and remove visible seams. Multi-band Blending technique was used for image blending [55]. This technique uses Laplacian Pyramid with Gaussian Kernel to blend the images while keeping their significant features. The images are reduced to different levels with the Gaussian Kernel. Afterwards, a Laplacian Pyramid is created, calculated based on the subtraction between the Gaussian image and the expansion of the Gaussian pyramid lower level. The Laplacian pyramids are then mixed, resulting in a final panorama obtained by interpolating and adding all of the pyramid’s levels [55].

### 2.6. Comparison Method

In order to evaluate the impact of using metaheuristics to improve the optimization problem of minimizing re-projection error in 360° panorama image stitching, the dispersion of final solutions, computational time and convergence curves are taken into consideration. In addition, an Analysis of Variance (ANOVA) and Tukey’s Honest Significance(THS) Tests were performed to statistically evaluate the validity of the simulation results. Before each run, a novel group of initial solution is generated. As a way to ensure that the final result of the optimization will achieve a result at least as good as the previously known acquisition parameters, the latter are used as the first individual of the initial population. The rest of the population is generated randomly within the search space according to Equation (34).

(34) $$X_{i,d}={\left[r\right]}_{0}^{1}\times \left(b_{d_{up}}-b_{d_{lo}}\right)+b_{d_{lo}};$$

**Figure 3:**
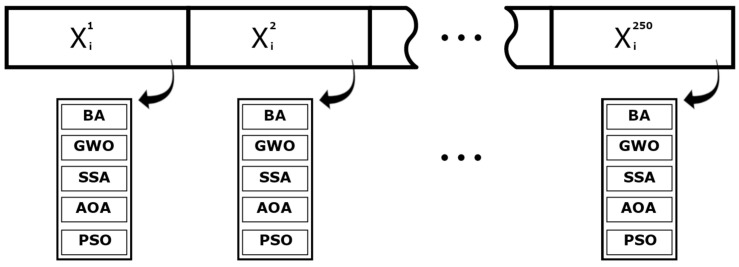
Database generation procedure.

Then, all metaheuristic algorithms run with the same initial group to avoid starting-point bias interfering with the comparison. All methods are population based and the population is set to 35. In addition, the stopping criteria for all algorithms is set as 1000 iterations. In that regard, the total number of objective function evaluations per method is equal. This is meant to ensure that the algorithms return a final solution at least as good as the one that was acquired initially. This procedure is illustrated by Figure 3.

Besides the population size and the maximum number of iterations, the techniques have other parameters and some are determined empirically. These parameters and definitions are presented in Table 1.

## 3. Results and Discussion

This section presents the obtained results for each proposed optimization method, and some discussions about them. The simulations were conducted by using an Intel Core i7-7700HQ CPU 2.80 GHz computer with 16 GB of RAM and Windows 10 64-bit operating system. The proposed method was implemented in C++ programming language.

**Figure 4:**
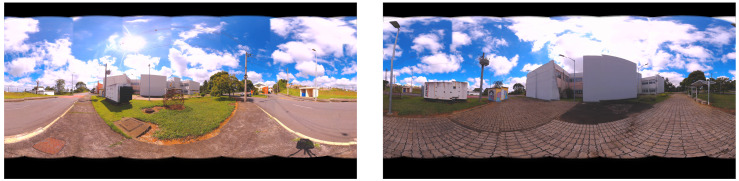
Arts and Design School at the Federal University of Juiz de Fora: Two point of View.

The proposed approaches are applied to image data from the Arts and Design School at the Federal University of Juiz de Fora, Brazil, from two different points of view, and compared with the Levenberg–Marquardt methodology. Figure 4 presents two points off view generated from the original image acquisition data.

### 3.1. Case Study I

The performance of BA, GWO, AOA, SSA and PSO are compared with respect to: (i) the final value of the objective function; (ii) convergence; (iii) boxplots of the results and (iv) statics indices. In addition, the results are analyzed with respect to that obtained by the classical Levenberg–Marquardt method.

**Figure 5:**
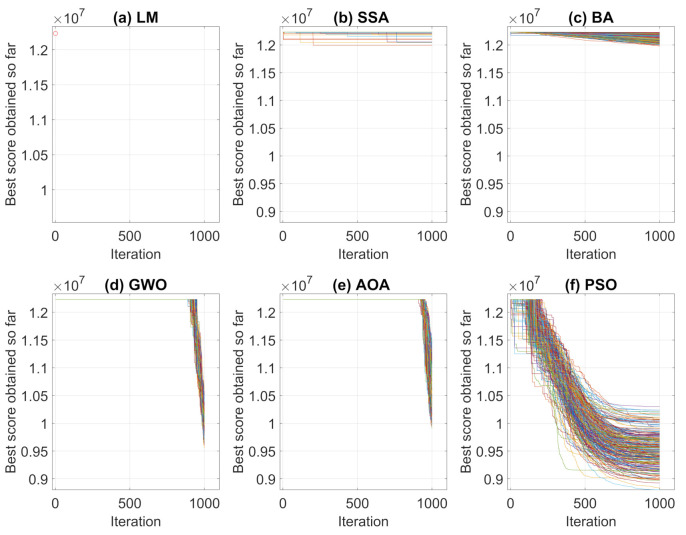
Convergence Curves in 250 simulations for Case Study I.

**Figure 6:**
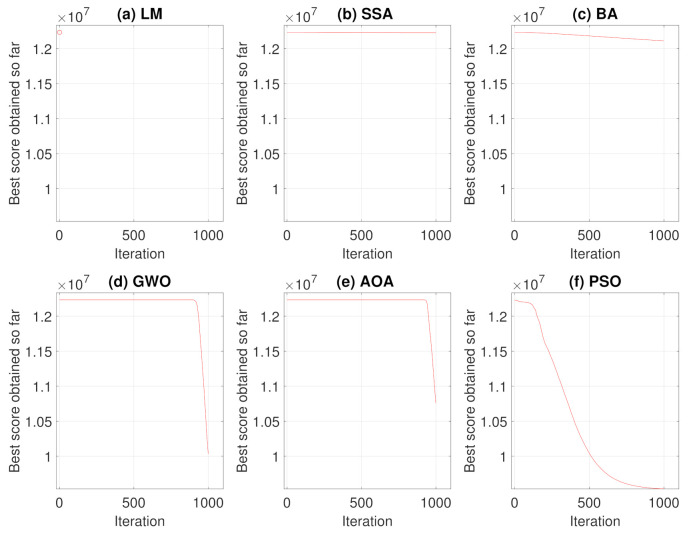
Average Convergence Curve.

Performing the algorithms described in Section 2, convergence curves and average convergence for the metaheuristic algorithms were obtained as shown in Figure 5 and Figure 6. Figure 5 shows the convergence history of each algorithms for the 250 simulations performed and Figure 6 shows the average curve of all convergence curves from all simulations.

Analyzing the responses obtained by the convergence curves presented in Figure 5 and Figure 6, it can be seen that in Levenberg–Marquardt method, the initial point interferes with the final solution, and as the initial guess is equal to the solution acquired by the robot, the method converged to the same solution, i.e., the solution presented is already a local optimum point. Therefore, the presented metaheuristics obtained a better response, since they found new solutions with objective function values of less than the initial one. When comparing the metaheuristics, the techniques convergence verifies that PSO and BA present a better global search stage compared to the others methods. That is, at process beginning, the algorithms perform an exploration of solution region and find good solutions. Meanwhile, GWO and AOA obtained similar convergence characteristics by having their differential in the local search step.

**Figure 7:**
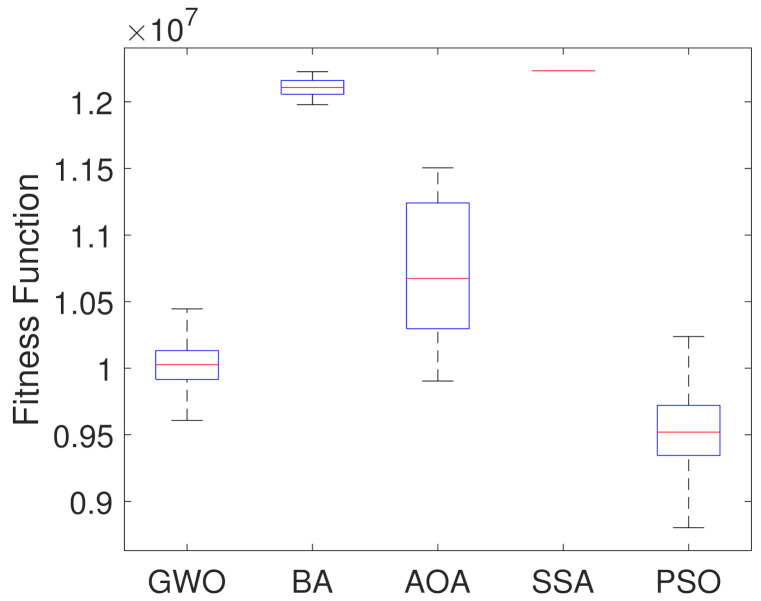
Boxplots of the Optimal Fitness Values from the 250 Simulations.

For further analysis, boxplot test is carried out for all the considered algorithms. They are presented in Figure 7, showing the objective function optimal values considering a set of 250 simulations. Table 2 presents statistical indices like median, mean and standard deviation of the fitness value acquired. They can be used to explain the information enclosed in the boxplot figure, and also present the average time (computational effort) in seconds of each algorithm.

Based on results in Figure 7 and Table 2, it can be noticed that PSO Algorithm outperforms the other metaheuristic algorithms in the optimization by presenting the lowest median, mean and fitness values. On the other hand, SSA showed the worst results. Although the Bat algorithm presents the second highest median value, it presents a smaller dispersion of the data, which indicates stability in the results, while the Arithmetic Optimization Algorithm presents greater variation. Table 2 indicates that the computation time values of all algorithms are similar to each other.

**Figure 8:**
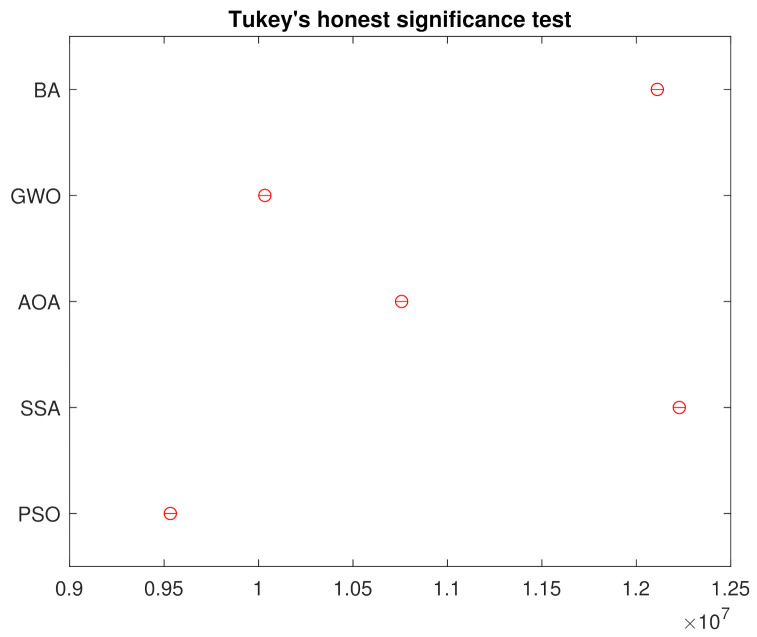
Tukey’s Honest Significance Test from the 250 Simulations.

In order to reject the null hypothesis, a variance test analysis is done, with results are shown in the Table 3. For further comparison, a Tukey’s honest significance was also done and the results in Figure 8 assures that the groups are all different from each other.

**Figure 9:**
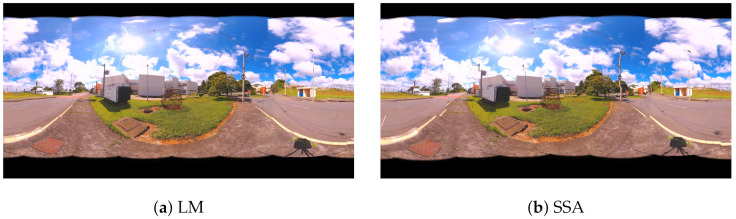

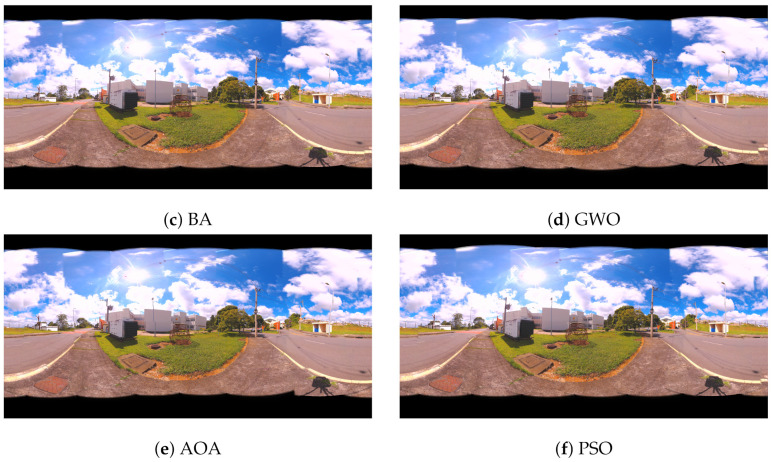
360 Panoramic Images Resulting from the Proposed Metaheuristics for Case Study I: (**a**) Levenberg–Marquardt (LM), (**b**) Salp Swarm Algorithm (SSA), (**c**) Bat Algorithm (BA), (**d**) Grey Wolf Optimizer (GWO), (**e**) Arithmetic Optimization Algorithm (AOA) and (**f**) Particle Swarm Optimization (PSO).

**Figure 10:**
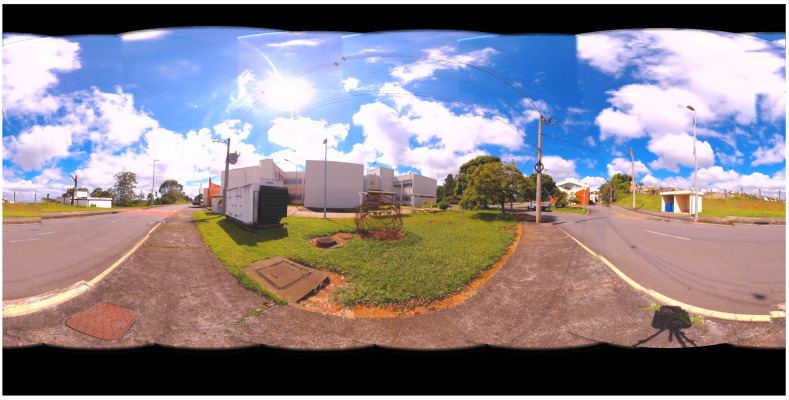
360 Panoramic Image Resulting from the best method for Case Study I: Particle Swarm Optimization (PSO).

Panoramic images are obtained from the values found by the algorithms. In Figure 9, the panoramas generated by the results of LM, AOA, GWO and SSA methods are shown. Figure 10 illustrates the 360 panorama that obtained the best solution found by the PSO Algorithm.

**Figure 11:**
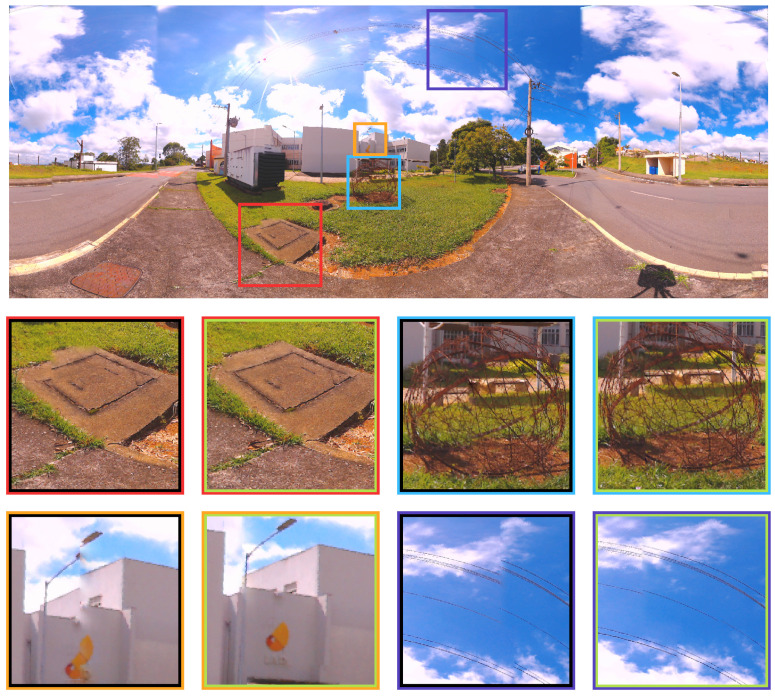
Zoomed figures in four spots for Case study I, represented by the red, cyan, yellow and dark blue squares. The differences of each approach for these spots are shown below in the image.

For a clearer visualization, some improvements in the objective function can be visualized in the zoomed figures of the artifacts present in the comparison between the LM (inner black square) and PSO (inner green square) optimized images in Figure 11. Four spots were chosen to represent the difference (red, cyan, yellow and dark blue squares). The details are shown below in the same figure.

Even with these improvements, the Panorama image is still not perfect and show some artifacts and misalignment because of the multimodal nature of the Bundle adjustment problem. More image processing procedures can further be used to enhance this panorama’s results. Although there is clear statistical improvement of the objective function, the RGB image still can be modified to become a more visually appealing panorama. In that regard, more procedures could further be used to enhance the panorama’s results and are still under investigation.

### 3.2. Case Study Ii

**Figure 12:**
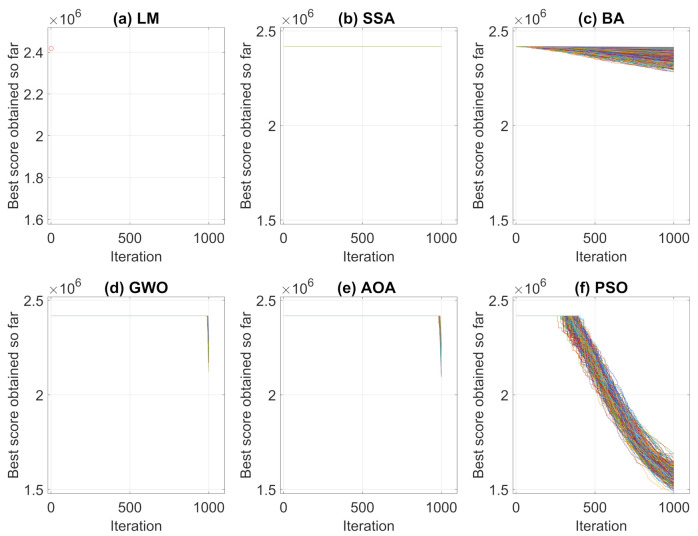
Convergence Curves in 250 simulations for Case Study II.

**Figure 13:**
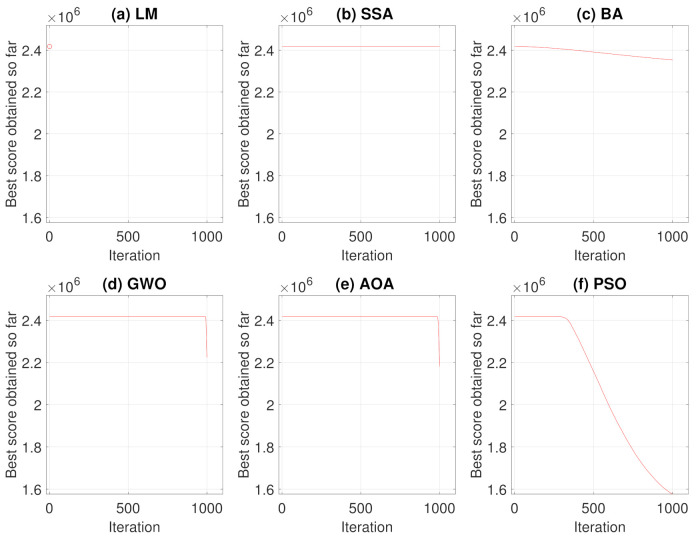
Average Convergence Curve.

The same analyses of Case Study I, presented in Section 3.1, were performed for Case Study II. Therefore, the results of convergence and average convergence curve of the methods can be seen in Figure 12 and Figure 13.

For this case, Levenberg–Marquardt’s behavior converging to the initial local minimum solution is also notorious. Analyzing the convergence curves of the other methods, it is observed that, the Particle Swarm Optimization and Bat techniques present a better global search in the solution, while Grey Wolf Optimizer and Arithmetic Optimization Algorithm have their prominence in the local search. The Salp Swarm Algorithm in all simulations, on the other hand, kept the fitness value equal to the original, that is, the algorithm did not find better solutions during iterations.

**Figure 14:**
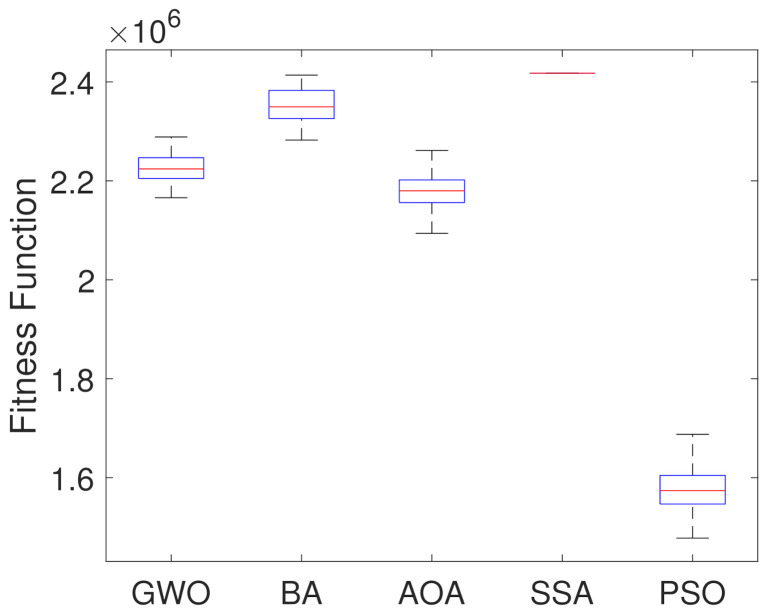
Boxplots of the optimal fitness values from the 250 simulations.

Figure 14 presents the boxplots of all methods considering the minimum values found in the 250 simulations. Table 2 presents the statistical data for better results analysis obtained by the boxplots and the average computational efforts in seconds for each algorithm.

Analyzing the results of the Figure 14 and Table 4, it can be seen that the PSO Algorithm presented the smallest objective function value as well as median and mean, but in relation to the others it presented the largest standard deviation. The Grey Wolf optimizer presented the smallest dispersion of the data. On the other side, Salp Swarm Algorithm performed the worst result, not improving the solution. Table 4 indicates that the computational time values of all algorithms are similar to each other.

**Figure 15:**
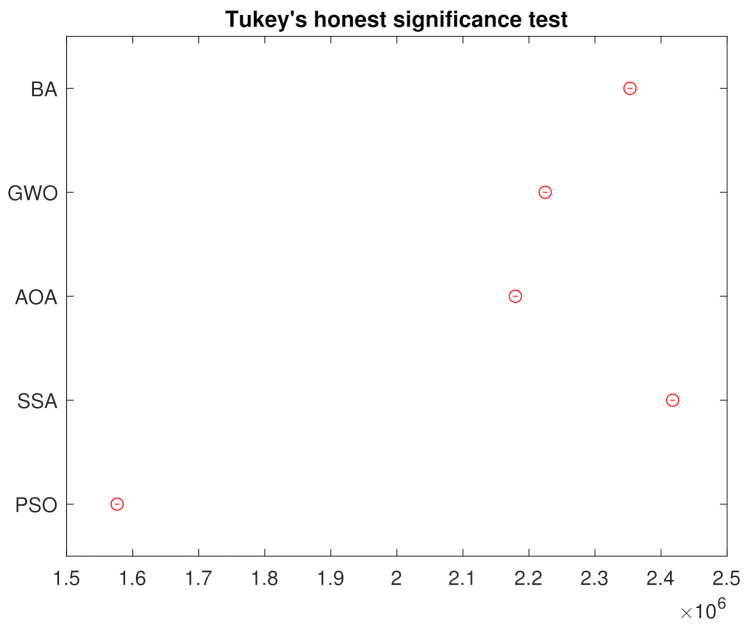
Tukey’s Honest Significance Test from the 250 Simulations.

In order to reject the null hypothesis, an analysis of variance test is done, and the results are shown in the Table 5. For further comparison, a Tukey’s honest significance was also done and the results in Figure 15 assures that the groups are all different from each other.

#### Panorama Results

**Figure 16:**
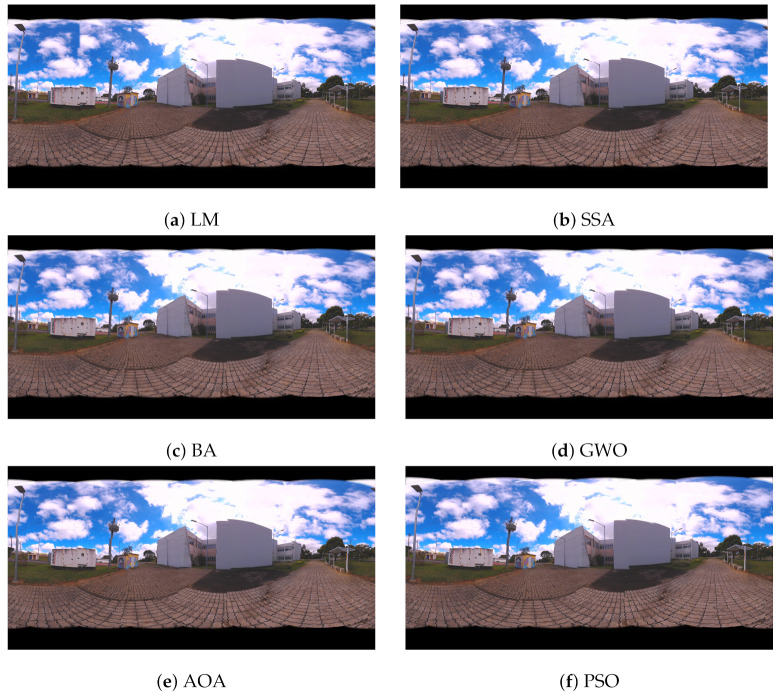
360 Panoramic Images Resulting from the Proposed Metaheuristics for Case Study II: (**a**) Levenberg–Marquardt (LM), (**b**) Salp Swarm Algorithm (SSA), (**c**) Bat Algorithm (BA), (**d**) Grey Wolf Optimizer (GWO), (**e**) Arithmetic Optimization Algorithm (AOA) and (**f**) Particle Swarm Optimization (PSO).

**Figure 17:**
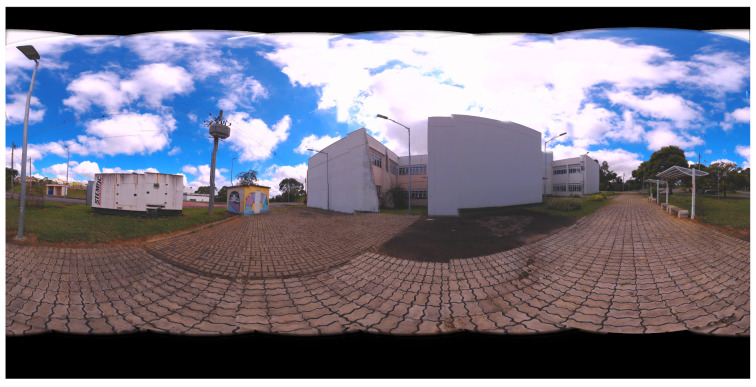
360 Panoramic Image Resulting from the best method for Case Study II : Particle Swarm Optimization (PSO).

The results of optimization algorithms are shown in Figure 16 and Figure 17 by means of the panoramic images generated. Figure 16 presents the panoramic results of LM, SSA, BA, GWO, AOA and PSO methods. Figure 17 illustrates the 360 panorama that obtained the best solution found by PSO Algorithm.

**Figure 18:**
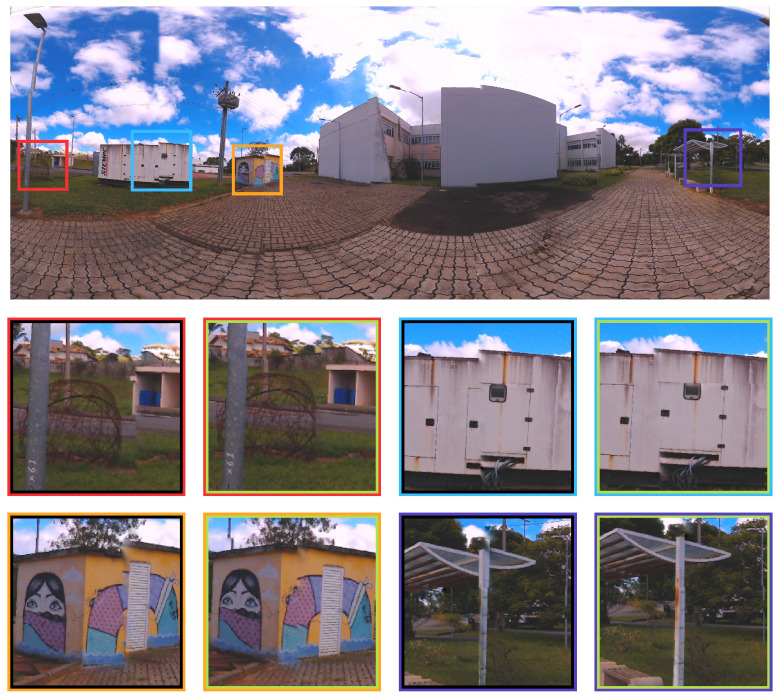
Case study II - Zoomed figures of four different spots represented by the red, cyan, yellow and dark blue squares show the nuance of each optimization approach.

For a clearer visualization, some improvements in the objective function can be visualized in the zoomed figures of the artifacts present in the comparison between the LM (inner black square) and PSO (inner green square) optimized images in Figure 18.

Although the panorama improved, the image is still not perfect and show some artifacts and misalignment that might be caused, in the same way as in case I, by the multimodal nature of the Bundle adjustment problem. Even though there is statistical improvement of the objective function, the RGB image still has room for improvement. In addition, more procedures could further be used to enhance the panorama’s results and are still under investigation.

For a more comprehensive visual comparison, an animated figure is available in the following link: https://github.com/PvirtualGit/Sensors2021 (accessed on 2 June 2021).

## 4. Conclusions and Future Work

The proposed research work presented the realization of bundle adjustment optimization through metaheuristics for 360 panoramic image generation. The main idea is to perform camera parameters optimization in order to achieve better alignment between images, comparing bio-inspired algorithms to the classical, derivative based, Levenberg–Marquardt used in the literature. The process was formulated as a problem to minimize the re-projection error, in order to find the best transformation that aligns the images to be stitched together.

The proposed techniques were evaluated in real scenarios and show good statistical results, proving an improvement in the solutions over the classical method, i.e., the metaheuristics obtained an improvement in the bundle adjustment problem. Although the panorama images are still not perfect, the objective of this study was achieved as the bio-inspired algorithms obtained better results than those found by Levenberg–Marquardt.

For future works, the modeling of hybrid algorithms can be explored in order to aggregate the main advantages of each chosen technique. Also, the image processing might be improved using the gain compensation described in [17] and a weighted average of the matches cost in the objective function, both of which are under evaluation by the time this article was written.

## Figures and Tables

**Table 1:** Parameter values for the comparative algorithms.

Algorithm	Parameter	Value
BA	A	1
	r	1
	λ	0.01
	α	0.9995
	$fr_{min}$	0
	$fr_{max}$	100
GWO	Convergence parameter (*a*)	Linear reduction from 2 to 0
AOA	α	5
	μ	0.49999
SSA	Convergence parameter ($C_{1}$)	According to Equation (20)
PSO	Topology	Global
	Cognitive and social constants	($C_{1},C_{2}$) 2,2
	Inertia weight	Linear reduction 0.9 to 0.2
	Velocity limit	6

**Table 2:** Comparison of Fitness Values Obtained by Metaheuristics and Average Execution Time.

Algorithm	Minimum	Median	Mean	Standard Deviation	Average Time (s)
GWO	$9.552480\;\times \;10^{6}$	$1.0027000\;\times \;10^{7}$	$1.0033\;\times \;10^{7}$	$1.7718\;\times \;10^{5}$	10.6541
BA	$1.1979000\;\times \;10^{7}$	$1.2106950\;\times \;10^{7}$	$1.2111\;\times \;10^{7}$	$6.4233\;\times \;10^{4}$	9.1809
AOA	$1.1504700\;\times \;10^{7}$	$1.0674300\;\times \;10^{7}$	$1.0757\;\times \;10^{7}$	$4.9586\;\times \;10^{5}$	9.7178
SSA	$1.1994400\;\times \;10^{7}$	$1.2233100\;\times \;10^{7}$	$1.2228\;\times \;10^{7}$	$2.8482\;\times \;10^{4}$	9.0267
PSO	$8.802380\;\times \;10^{6}$	$9.520155\;\times \;10^{6}$	$9.5329\;\times \;10^{6}$	$2.9654\;\times \;10^{5}$	9.2606

**Table 3:** Anova Table.

Source	SS	df	MS	F	P > F
**Treatments**	$1.466\;\times \;10^{15}$	4	$3.665\;\times \;10^{14}$	49,951.36	0
**Error**	$9.216\;\times \;10^{13}$	1245	$7.403\;\times \;10^{10}$	-	-
**Total**	$1.558\;\times \;10^{15}$	1249	-	-	-

**Table 4:** Comparison of fitness values obtained by metaheuristics and average execution time.

Algorithm	Minimum	Median	Mean	Standard Deviation	Average Time (sec)
GWO	$2.119770\;\times \;10^{6}$	$2.224145\;\times \;10^{6}$	$2.2247\;\times \;10^{6}$	$2.9250\;\times \;10^{4}$	15.1934
BA	$2.282500\;\times \;10^{6}$	$2.349620\;\times \;10^{6}$	$2.3530\;\times \;10^{6}$	$3.3987\;\times \;10^{4}$	13.7660
AOA	$2.179840\;\times \;10^{6}$	$2.179840\;\times \;10^{6}$	$2.1794\;\times \;10^{6}$	$3.3183\;\times \;10^{4}$	14.3220
SSA	$2.417660\;\times \;10^{6}$	$2.417660\;\times \;10^{6}$	$2.417660\;\times \;10^{6}$	0	13.4370
PSO	$1.477820\;\times \;10^{6}$	$1.573870\;\times \;10^{6}$	$1.5764\;\times \;10^{6}$	$4.0016\;\times \;10^{4}$	13.6258

**Table 5:** Anova Table.

Source	SS	df	MS	F	P > F
**Treatments**	$1.120\;\times \;10^{14}$	4	$2.802\;\times \;10^{13}$	29,726.08	0
**Error**	$1.174\;\times \;10^{12}$	1245	$9.426\;\times \;10^{8}$	-	-
**Total**	$1.132\;\times \;10^{14}$	1249	-	-	-

## Data Availability

No available data.
